# Continuous Evolution of Eu^2+^/Eu^3+^ Mixed Valency Driven by Pressure and Temperature

**DOI:** 10.1021/acs.jpca.4c08660

**Published:** 2025-02-25

**Authors:** Mingyu Xu, Greeshma C. Jose, Mouyang Cheng, Cheng Peng, Jose L. Gonzalez Jimenez, Wenli Bi, Mingda Li, Weiwei Xie

**Affiliations:** † Department of Chemistry, Michigan State University, East Lansing, Michigan 48824, United States; ‡ Department of Physics, University of Alabama, Birmingham, Alabama 35294, United States; § Quantum Measurement Group, Massachusetts Institute of Technology, Cambridge, Massachusetts 02139, United States; ∥ Department of Materials Science and Engineering, Massachusetts Institute of Technology, Cambridge, Massachusetts 02139, United States; ⊥ Center for Computational Science & Engineering, Massachusetts Institute of Technology, Cambridge, Massachusetts 02139, United States; # Department of Nuclear Science and Engineering, Massachusetts Institute of Technology, Cambridge, Massachusetts 02139, United States

## Abstract

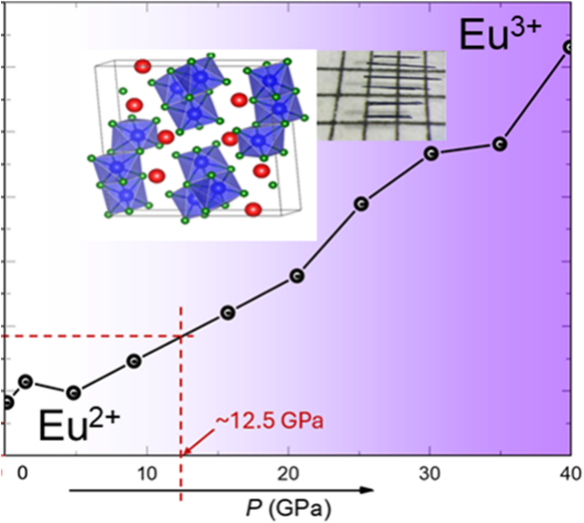

Continuous mixed valency involving Eu^2+^ and Eu^3+^ in Eu_4_Bi_6_Se_13_ can be induced under applied pressure or at reduced temperatures. The monoclinic structure of Eu_4_Bi_6_Se_13_, crystallizing in the *P*2_1_/*m* space group (No. 11), features linear chains of Eu atoms aligned along the *b*-axis. Magnetic susceptibility measurements, conducted both parallel and perpendicular to the *b*-axis and analyzed using Curie–Weiss theory, alongside high-pressure partial fluorescence yield (PFY) data from X-ray absorption spectroscopy (XAS), indicate the material’s propensity to adopt a mixed-valent state. Within this state, the trivalent Eu^3+^ configuration becomes increasingly favored as the pressure rises or the temperature decreases.

## Introduction

Rare-earth intermetallic compounds exhibit a diverse range of magnetic phenomena, making them crucial in the study of quantum materials.^1^ Unstable 4f electrons and mix valency (e.g., Ce, Sm, Eu, Tm, Yb) display notable quantum behaviors such as the Kondo effect,^2^ valence fluctuations,^3^ unconventional superconductivity,^4^ non-Fermi-liquid states,^5−7^ and critical fluctuations near quantum critical points^8−10^ in the rare-earth compounds. In these systems, the localized 4f electrons, influenced by strong Coulomb interactions, can hybridize with the extended conduction band of itinerant *s*-*d* electrons.^5,11^ The fluctuating valence state appears when the energy difference between competing valence states is small, which is even smaller than the f-electron bandwidth.^12^ The resulting electronic structure and magnetic behavior are highly dependent on hybridization strength, band structure, and electronic configuration. Non-Fermi-liquid behaviors, for instance, typically emerge in regions where long-range magnetic order is destabilized by spin fluctuations or other quantum critical phenomena.^13^ All of these make the study of valence changes in rare-earth compounds highly significant in studying quantum phenomena. Research in this field has primarily focused on Ce-, Yb-, and Eu-based heavy-Fermion systems, particularly in families such as RT_2_X_2_ (where T is a 3d or 4d transition metal, and X is Si or Ge). Compounds like CeCu_6–*x*_Au*_x_* exhibit a strong interplay between long-range magnetic order and Kondo fluctuations,^14−16^ offering valuable insights into novel quantum critical behaviors. Though previous studies have shown that Eu^2+^ in EuSn_2_P_2_^17^ and EuCd_2_As_2_^18^ remains remarkably stable under high pressures up to 40 GPa, many Eu-based materials exhibit pressure-sensitive valence states and mixed valency.^19−22^ This indicates that investigating the valency of Eu compounds is essential for uncovering and understanding unique quantum phenomena.

Valence instability not only appears in single-electron (or -hole) occupancies in their 4f shells but also in rare-earth elements with multiple 4f-shell occupancies, such as Sm, Eu, Tm, and potentially Pr, and is observed in some well-known Kondo insulators, including SmB_6_.^23,24^ These findings raise intriguing questions about whether spin fluctuations can serve as indicators of mixed valence states under external conditions, such as temperature, pressure, and magnetic fields. Indeed, Eu-based 1–2–2 compounds have been shown to exhibit unconventional behaviors such as hybridization gap formation in EuNi_2_P_2_,^25^ reentrant superconductivity under pressure in EuFe_2_As_2_, where long-range Eu magnetic order competes with superconductivity, and valence instability in Eu.^26^ However, these phenomena are rarely reported in other Eu-based systems.

Recently, our group discovered a new compound, Eu_4_Bi_6_Se_13_, which exhibits spin fluctuation, spin-flop, and metamagnetic transition.^27^ This observation led us to hypothesize that Eu4Bi6Se_13_ might exhibit mixed valence behavior under variable temperatures and pressures.^28−30^ To investigate this, we performed Curie–Weiss fitting on the magnetic susceptibility data and conducted high-pressure X-ray absorption spectroscopy (XAS) experiments to study the temperature- and pressure-dependent valence states of Eu in Eu_4_Bi_6_Se_13_. The Curie–Weiss analysis of magnetic susceptibility, measured both perpendicular and parallel to the *b*-axis, and high-pressure partial fluorescence yield (PFY) data from X-ray absorption spectroscopy (XAS) reveal a tendency for the material to adopt a mixed-valent state. In this state, the trivalent Eu configuration becomes more pronounced as the pressure increases or temperature decreases.

## Experimental Methods and Calculation Details

### Phase Identification

The needled-shaped single crystal was synthesized from the solid-state pellet reaction. Europium pieces (sublimed dendritic, REO grade, sourced from Thermo Scientific), bismuth pieces (purity of 99.99%, provided by Strem Chemicals Inc.), and selenium powder (with a purity of 99.995%, obtained from Thermo Scientific) with 4:6:13 ratio were homogeneously mixed, pressed into pellets, put in an alumina crucible, and sealed in evacuated quartz tubes. The sample was heated to 800 °C at a ramp rate of 60 °C/h, maintained at 800 °C for 48 h, and then cooled at the same rate to room temperature. Given a small amount of needle-like crystals and the significant background interference from laboratory Cu Kα radiation, high-pressure phase analysis uses synchrotron powder X-ray diffraction. This was obtained from pulverized needle-like crystals at the 13BM-C beamline (PX2) of the Advanced Photon Source (APS) at Argonne National Laboratory (ANL), employing a wavelength of 0.434 Å. A BX-90 Diamond Anvil Cell, equipped with a 200 μm culet, was utilized to apply the pressure. A 4:1 (volume ratio) methanol–ethanol mixture was used as the pressure-transmitting medium.^22^ The two-dimensional diffraction images were integrated using Dioptas software, and Rietveld refinement of the data set was conducted with GSAS II.^23^

### Heat Capacity Measurements

Temperature-dependent specific heat measurements were carried out using the relaxation technique as implemented in the heat capacity option of the quantum design, physical property measurement system (PPMS-DynaCool).

### High-Pressure Valence State Determination

To provide direct information on the valence of Eu in the compound under pressure, the PFY-XAS (partial fluorescence yield X-ray spectrometry) experiment at the L_3_ edge (6.974 keV) was conducted at room temperature at Beamline 161D-D of the APS, ANL. The beam size was focused on a 4 × 6 μm^2^ area. The incident X-ray energy was scanned from 6.956 to 7.032 keV. To avoid strong absorption by the diamond anvils, incoming X-rays traveled through a Be gasket, and the fluorescence signal was collected. The signal was first analyzed with a silicon analyzer and then collected with a Pilatus detector at 90 °C from the incident X-ray beam. Since the measurements were conducted in fluorescence mode, attenuation of fluorescence may occur as it travels out of the sample. The optimal sample position was determined by scanning the sample position to minimize this attenuation. High pressure was achieved using a symmetric-type diamond anvil cell equipped with a pair of diamond anvils with a 300 μm culet. A beryllium gasket was preindented to ∼50 μm, and a hole of 200 μm was drilled. Cubic boron nitride (cBN) and epoxy mixture were then filled in the gasket hole and indented to form the gasket insert and maintain a good sample thickness under pressure. A 40 × 70 μm^2^ Eu_4_Bi_6_Se_13_ needle was loaded into the sample chamber formed by the cBN insert along with a ruby ball used as an in situ pressure marker. No pressure medium was used. Data was taken under compression up to 40 GPa and then under decompression to 2.2 GPa, with one point taken at the lowest pressure under decompression. Data was normalized in Athena, a part of the Demeter software package for XAS data processing and analysis.^24^ Eu mean valence was obtained by modeling the XAS spectrum with two sets of Lorentzian and arctangent functions for divalent and trivalent states.

## Results and Discussion

**Figure 1:**
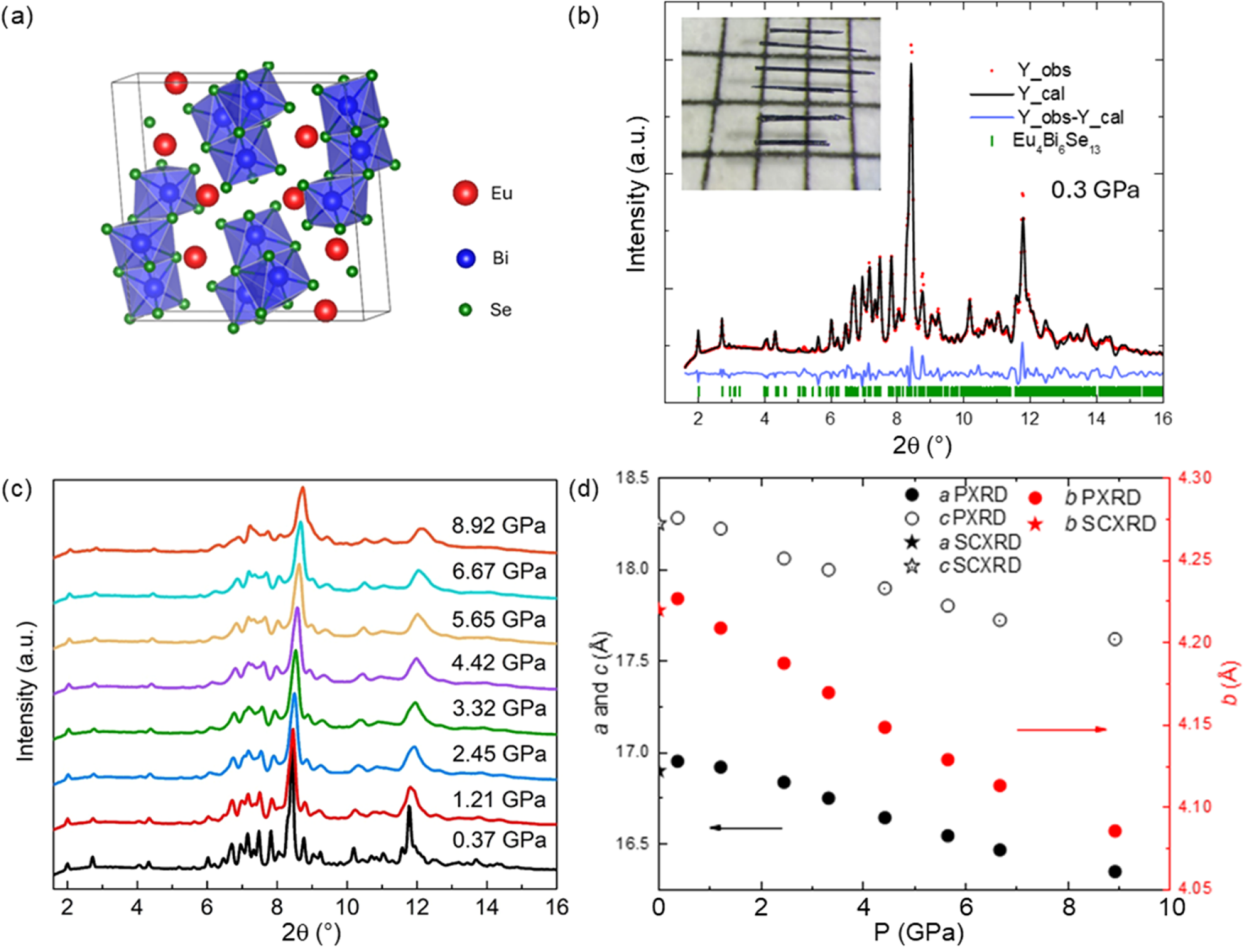
Crystal structure of Eu_4_Bi_6_Se_13_ and high-pressure powder X-ray diffraction measurements. (a) Crystal structure of Eu_4_Bi_6_Se_13_ showing the edge-share distorted BiSe_6_ octahedra. (b) Powder X-ray diffraction (PXRD) data were obtained at 0.3 GPa. The inset shows the picture of crystals on the millimeter grid paper. (c) Powder X-ray diffraction patterns were collected at room temperature at various pressures of up to 9 GPa. (d) Lattice parameters comparison between single-crystal X-ray diffraction (SCXRD) and PXRD under high pressure.

Eu_4_Bi_6_Se_13_ crystallizes in the monoclinic *P*2_1_/*m* space group, sharing isostructural characteristics with Sr_4_Bi_6_Se_13_. As illustrated in Figure 1a, the crystal structure was confirmed via single-crystal X-ray diffraction refinement,^27^ revealing edge-sharing distorted BiSe_6_ octahedra that form extended chains and block-like motifs. Europium atoms are embedded within these octahedral layers and are positioned near the bismuth sites. The Eu–Eu interactions establish linear chains along the *b*-axis, with a uniform Eu–Eu bond distance corresponding to the unit cell parameter of 4.219(1) Å. All of the atoms sites in the same ac plane (as shown in Table S1), which forms the pseudo-2-dimensional structure. This structure increases compressibility, and the complicated environment of Eu enhances the valence change. Phase purity was confirmed by powder Le Bail refinement using synchrotron X-ray data obtained at the Advanced Photon Source (APS) at Argonne National Laboratory (ANL), as shown in Figure 1b. The pressure-dependent X-ray diffraction patterns in Figure 1c confirm the absence of any structural phase transition in Eu_4_Bi_6_Se_13_ up to 9 GPa. Figure 1d depicts the evolution of lattice parameters *a*, *b*, and *c* with applied pressure, refined from powder X-ray diffraction (PXRD) data. The lattice parameters exhibit a continuous decrease with increasing pressure. This steady contraction of the lattice parameters further corroborates the lack of a phase transition within the examined pressure range.

**Figure 2:**
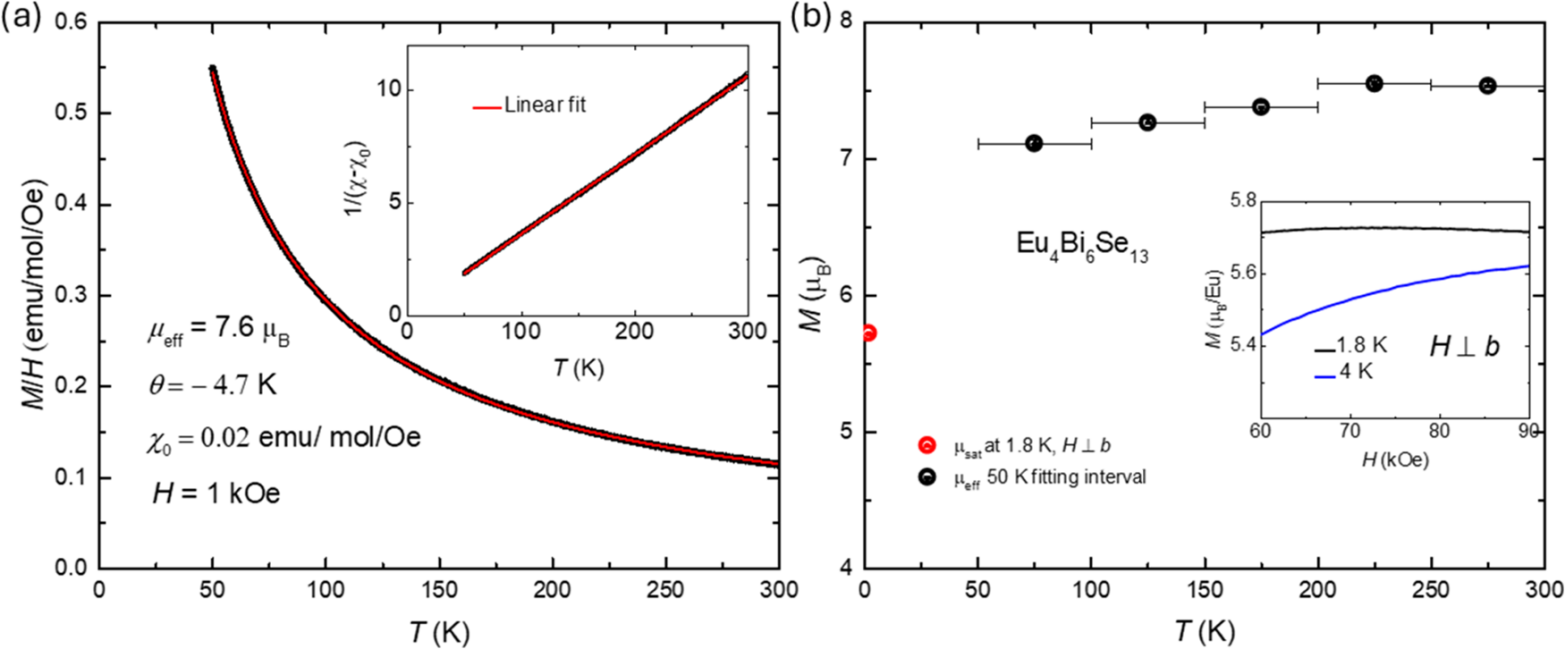
Temperature-dependent mixed valency in Eu_4_Bi_6_Se_13_ (a) Curie–Weiss (CW) fitting on the polycrystalline average susceptibility with the range of temperature 50 K–300 K calculated as χ = (2χ_⊥_ + χ_∥_)/3, where χ_⊥_ and χ_∥_ denote susceptibilities perpendicular and parallel to the crystallographic *b*-axis, respectively. The inset shows the linear fitting. (b) Effective moments compared with saturation moments at 1.8 K. The inset shows the field-dependent magnetization.

The magnetic susceptibility measurements of Eu_4_Bi_6_Se_13_ were conducted at ambient pressure, revealing that the magnetic moments evolve continuously as the temperature decreases. Figure 2a shows the Curie–Weiss (CW) fitting with the temperature range of 50 K–300 K. The CW analysis was performed on the polycrystalline average susceptibility using a magnetic field of 1 kOe, calculated as χ = (2χ_⊥_ + χ_∥_)/3 (β is close to 90 °C, and the *a* and *c* lattice parameters are similar), where χ_⊥_ and χ_∥_ denote susceptibilities perpendicular and parallel to the crystallographic *b*-axis, respectively. The μ_eff_ is 7.57 μ_B_, slightly less but close to the theoretical values of 7.94 μ_B_ of Eu^2+^.^25^ Assuming that Eu^3+^ contributes zero effective moment (*J* = 0), the value of 7.57 μ_B_ corresponds to approximately 95% Eu^2+^ and 5% Eu^3+^. To further analyze the susceptibility, linear fitting was applied to the susceptibility data after subtracting the temperature-independent component (χ_0_) across the temperature range of 50–300 K in 50 K intervals, as shown in Figure 2b. In the temperature range of 250–300 K, the fitted μ_eff_ is 7.53 μB, consistent with the presence of ∼5% Eu^3+^ near room temperature. This finding aligns with the ∼8% Eu^3+^ observed at 0.1 GPa from PYF-XAS spectra analysis, which is discussed later in the content. Previous reports indicate that as the temperature decreases, the lattice parameters *a*, *b*, and *c* contract, as shown in Figure S1b, which also illustrates the temperature dependence of the angle β and unit cell volume. This result motivated us to investigate the volume changes associated with the mixed valency of Eu^2+^/Eu^3+^ in Eu_4_Bi_6_Se_13_.

**Figure 3:**
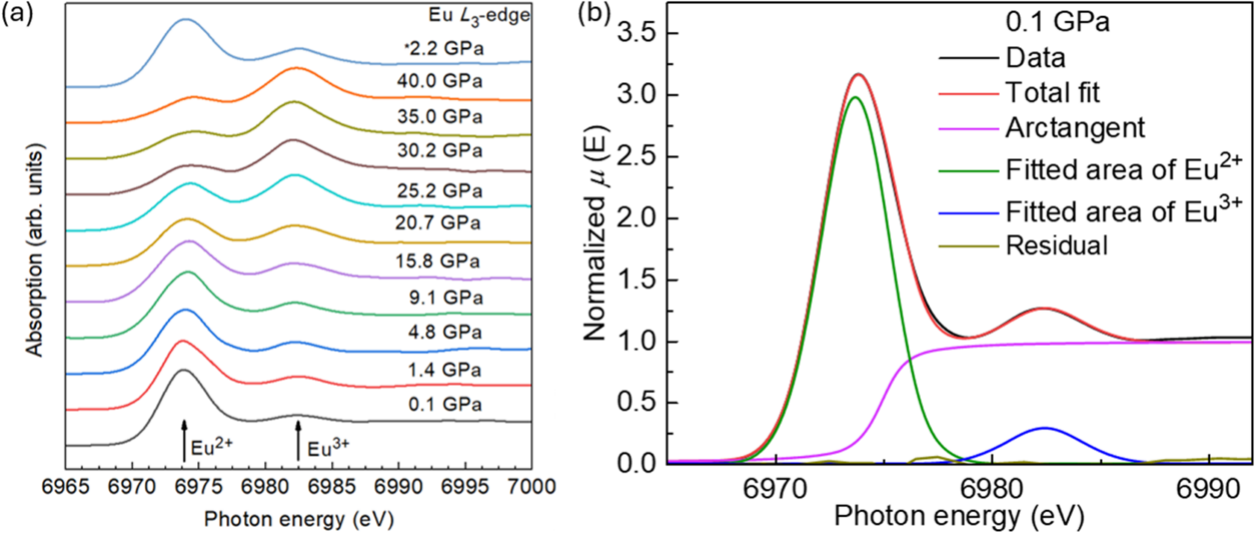
High-pressure partial fluorescence yield X-ray spectrometry (PYF-XAS) spectra and analysis. (a) PYF-XAS spectra at the *L*_3_ edge in Eu_4_Bi_6_Se_13_ with increasing pressure from 0.1 to 40.0 GPa along with a decompressed pressure of 2.2 GPa (marked with an asterisk). (b) Analysis of the PFY-XAS data at 0.1 GPa using two sets of Lorentzian and arctangent functions for Eu^2+^ and Eu^3+^.

To investigate the valencies of Eu under decreasing volumes in Eu_4_Bi_6_Se_13_, high-pressure PYF-XAS measurements were performed at room temperature. Figure 3a displays the PYF-XAS spectra of Eu_4_Bi_6_Se_13_ as a function of applied pressure. Data were collected with increasing pressure from 0.1 to 40.0 GPa, followed by decompression to 2.2 GPa. At 0.1 GPa, the Eu ions are predominantly in the divalent state, with approximately 10% in the trivalent state. This is evidenced by a dominant peak above 6.974 keV, corresponding to 2p_3/2_^6^4f ^7^5d^0^6s^2^ → 2p_3/2_^5^4f ^7^5d^1^6s^2^ transition, and a small absorption peak, appearing at ∼8 eV higher, attributed to 2p_3/2_^6^4f ^6^5d^1^6s^2^ → 2p_3/2_^5^4f ^6^5d^2^6s^2^ transition.^26^ As the pressure increases, the intensity of the Eu^2+^ absorption peak decreases, while the Eu^3+^ absorption peak intensity increases, indicating a valence transition from the divalent to the trivalent state. Upon decompression, the divalent state of Eu ions is recovered, demonstrating a reversible valence transition. The mean valence of Eu under pressure was determined by modeling the PFY-XAS data using a combination of Lorentzian and arctangent functions for each absorption peak, as illustrated in Figure 3b.

**Figure 4:**
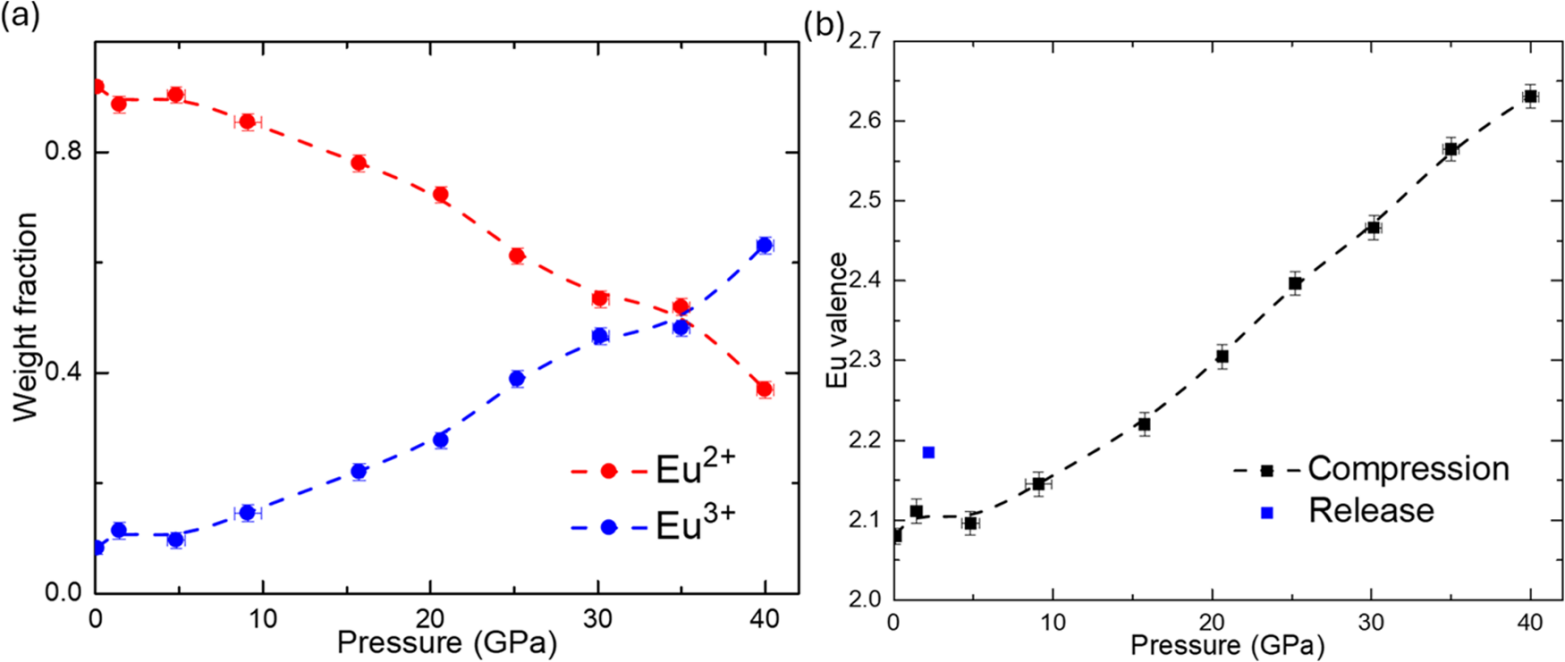
Weight fraction and Eu valence as a function of pressure from PYF-XAS spectra analysis. (a) Pressure-dependent weight fraction; (b) Eu valence measured using PYF-XAS spectra analysis. The blue point presents the average valence from releasing pressure.

The weight fractions of Eu^2+^ and Eu^3+^ as a function of pressure, derived from the PFY-XAS spectra, are shown in Figure 4a. As pressure increases, the fraction of Eu^2+^ decreases while that of Eu^3+^ increases. At 35 GPa, the ratio of Eu^2+^ to Eu^3+^ reaches approximately 50%, and with further pressure increases, the fraction of Eu^3+^ continues to rise. Figure 4b illustrates the Eu valence as determined from the fractions of Eu^2+^ and Eu^3+^. The black data points represent measurements taken during the pressure increase, while the blue data point corresponds to the measurement taken during the pressure decrease. After releasing the pressure, the valence returns to an Eu^2+^-rich state, confirming the reversible nature of the valence transition.

**Figure 5:**
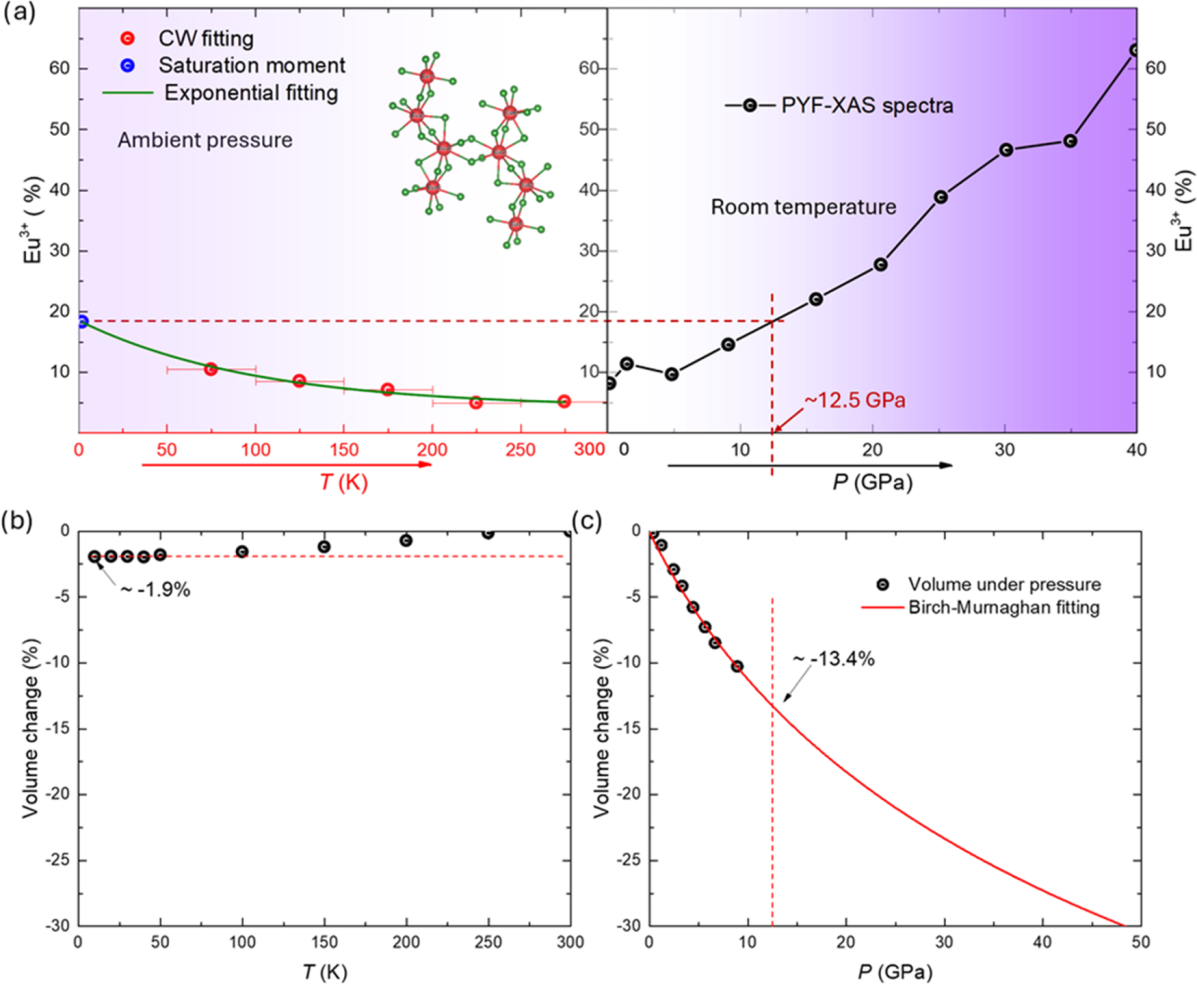
(a) Percentage of Eu^3+^ as a function of temperature at ambient pressure and as a function of pressure at room temperature fitted by CW fitting and field-dependent magnetization measurements at ambient pressure. The red symbols give the results from the CW fitting, and the blue symbol shows the Eu^3+^ ratio estimated using the saturation moment. The green line shows the exponential fitting. The right panel presents the Eu^3+^ ratio as a function of the pressure determined by PYF-XAS spectra at room temperature. The black symbols present the results from PYF-XAS. The color gradation indicates the change in the Eu^3+^ ratio. The arrows give the increase directions of the temperature and pressure. Volume change as (b) a function of temperature and (c) as a function of pressure with the pressure projected up to 47 GPa. The red solid line is Birch–Murnaghan fitting using *P* = 3B_0_/2[(*V*_0_/*V*)^7/3^ – (*V*_0_/*V*)^5/3^]. The bulk modulus of *B*_0_ is calculated to be 66.5 GPa.

Both increasing the pressure and decreasing the temperature reduce the unit cell volume, which can induce a transition from Eu^2+^ to Eu^3+^. Thus, to investigate the effects of volume changes on the mixed valency of Eu^2+^/Eu^3+^, the Eu^3+^ ratio was analyzed as a function of the temperature and pressure, as shown in Figure 5a. As the temperature decreases, the Eu^3+^ ratio increases. At the range of temperature of 50K −100 K, the ratio of Eu^3+^ increases to 11%. This increase is also shown in the inset of Figure 2b, and the saturation moment, at 1.8 K, is 5.72 μ_B_. Given the saturation moments of Eu^2+^ (7 μ_B_) and Eu^3+^ (0 μ_B_), this corresponds to an 18% Eu^3+^ fraction at 1.8 K. Fitting the 1.8 K data, alongside Curie–Weiss results, with an exponential function reveals a consistent trend in the Eu^3+^ ratio as temperature decreases. Figure 5b,c present the Eu^3+^ ratio as a function of the temperature and pressure. Compared to the measured range of pressure, the change in volume is much smaller in the temperature of 1.8 K −300 K. The 18% Eu^3+^ increase can also be induced by the 12.5 GPa pressure, which corresponds to a 13.4% volume difference. The valence change under pressure should be due to the different sizes of ionic radius of Eu^2+^ and Eu^3+^, and lattice parameters change under temperature is much smaller. All of these suggested that, in the low-temperature range, the valence of Eu is not dominantly affected by the cell volume changes, as shown by high-pressure measurements, but influenced by the electron structure, which is the hybridization of conduction election and 4f electrons. This also indicates that in this system, the 4f electron bands seem very close to the Fermi level; even hundreds of Kelvin can affect it (tens of meV instead of a few eV away from the Fermi level). Simply put, the 4f electron is not well localized in this system.

**Figure 6:**
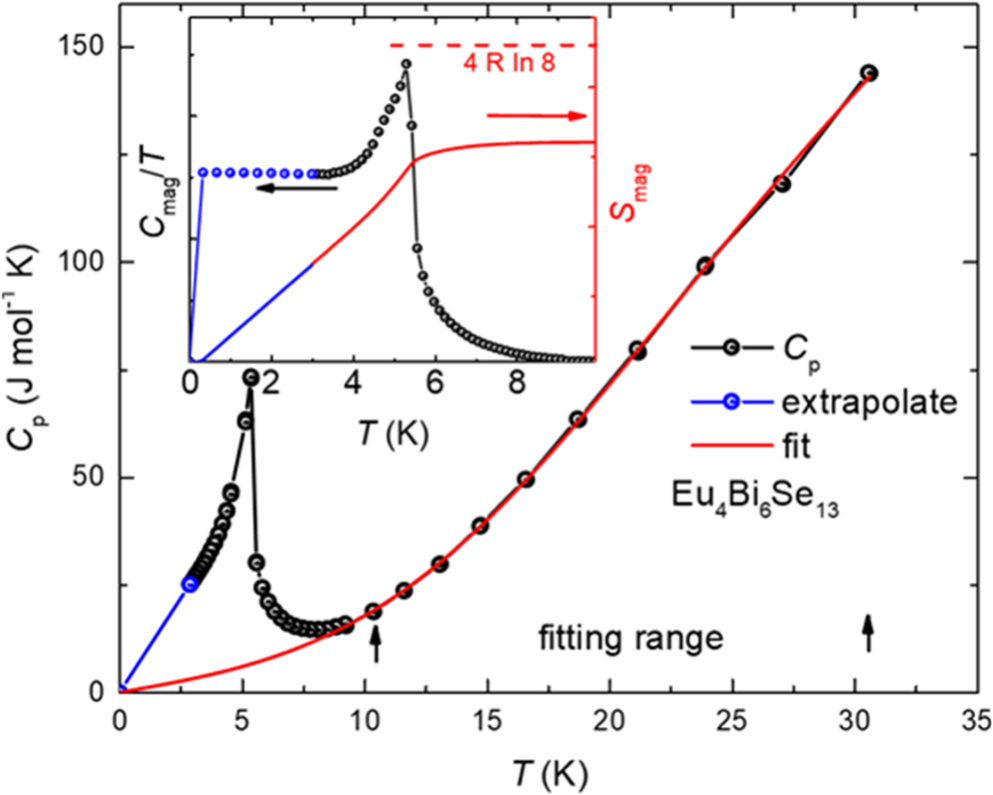
Specific heat fitting and magnetic entropy calculation (inset).

To verify that the transition is intrinsic to the system, heat capacity measurements were performed, and the magnetic contribution was calculated. Figure 6 illustrates the specific heat data fitted using the equation: *C*_p_= γ*T* + 9*NR*(*T*/Θ_D_)^3^∫_0_^Θ_D_/*T*^(*x*^4^ e^*x*^/(e^*x*^ – 1)^2^)d*x*. Here, γ represents the electronic contribution, *N* is the number of moles, *R* is the gas constant, and Θ_D_ is the Debye temperature. The blue points and lines in the figure represent extrapolated data. The inset shows the calculated magnetic specific heat and entropy. From the magnetic entropy, approximately 30% of Eu^3+^ is estimated to be present in the system. However, due to the limitations of the extrapolation in the magnetic specific heat data, this value may vary. Despite this uncertainty, the specific heat fitting results support the conclusion that the saturation moment observed in the field-dependent magnetic measurements represents the actual behavior of the system.

## Conclusions

In this study, we performed pressure- and temperature-dependent valence studies on the Eu atoms in Eu_4_Bi_6_Se_13_. PFY-XAS studies showcased a monotonic valency change toward a trivalent state with increasing pressure. This phenomenon is considered to be a result of the volume collapse of an intermediate valence state. The temperature-dependent valence change is discovered, which is shown using the CW fitting of the temperature-dependent magnetic susceptibility data, field-dependent magnetization, and magnetic specific heat analysis. This, which is different from pressure-dependent valence change, is further proved by temperature-dependent PXRD measurements. This valence change indicates the short distance of the 4f bands from the Fermi surface.

## Supporting Information Available

Atomic coordinates of Eu_4_Bi_6_Se_13_ single crystal; bonding length of Eu under pressure; β and volume as a function of temperature and pressure; Eu^3+^ percentage as a function of volume changes; SEM and EDS results; and magnetization measurements (PDF)
