# Supplementary material for: Continuous Evolution of Eu2+/Eu3+ Mixed Valency Driven by Pressure and Temperature
Source: J Phys Chem A. 2025 Feb 25;129(9):2371–7. doi: 10.1021/acs.jpca.4c08660 (PMC11891896; doi:10.1021/acs.jpca.4c08660)
Supplement: Supplementary file 1 — jp4c08660_si_001.pdf [file jp4c08660_si_001.pdf]

## Support Information

# Continuous Evolution of $Eu^{2+}/Eu^{3+}$ Mixed Valency Driven by Pressure and Temperature

*Mingyu Xu*[1], *Greeshma C. Jose*[2], *Mouyang Cheng*[3,4,5], *Cheng Peng*[1], *Jose L. Gonzalez Jimenez*[1], *Wenli Bi*[2], *Mingda Li*[3,6], *Weiwei Xie*[1*]

1. Department of Chemistry, Michigan State University, East Lansing, MI, 48864, USA
2. Department of Physics, University of Alabama, Birmingham, AL 35294, USA
3. Quantum Measurement Group, Massachusetts Institute of Technology, Cambridge, MA, 02139, USA
4. Department of Materials Science and Engineering, Massachusetts Institute of Technology, Cambridge, MA, 02139, USA
5. Center for Computational Science & Engineering, Massachusetts Institute of Technology, Cambridge, MA, 02139, USA
6. Department of Nuclear Science and Engineering, Massachusetts Institute of Technology, Cambridge, MA, 02139, USA

Corresponding Author: Weiwei Xie (xieweiwe@msu.edu)

### Table of Content

**Table S1.** Structure Refinement and atomic coordinates of $Eu_4Bi_6Se_{13}$ single crystal………..…S2

**Table S2.** Bonding length of Eu under pressure..……………….………………………………….S4

**Figure S1.** β and volume as a function of temperature and pressure……………………………S5

**Figure S2.** $Eu^{3+}$ percentage as a function of volume change……………………………………S6

**Figure S3.** SEM and EDS results ……………………...……..………………………………S7

**Figure S4.** Magnetization measurements…,,,,,…………………………………………………S8

**Table S1a.** Single crystal crystallographic data and structure refinement for $Eu_4Bi_6Se_{13}$ single crystal at 100 K. Values in parentheses are estimated standard deviations from refinement.

| **Refined Formula** | **$Eu_4Bi_6Se_{13}$** |
|---|---|
| F. W. (g/mol) | 2888.20 |
| Temperature (K) | 100.15 |
| Space group | $P2_1/m$ |
| *a* (Å) | 16.8965(2) |
| *b* (Å) | 4.2194(5) |
| *c* (Å) | 18.2479(2) |
| β (°) | 90.6664(12) |
| V (Å$^3$) | 1300.87(3) |
| Z | 2 |
| $2\theta$ range (°) | 5.05 to 84.534 |
| Density (calculated) | 7.373 g/cm$^3$ |
| Absorption coefficient | 68.082 mm$^{-1}$ |
| Reflections collected | 118175 |
| Independent reflections | 9991 [$R_{int}$ = 0.0822] |
| Refinement method | Full-matrix least-squares on $F^2$ |
| Data/restraints/parameters | 9991/0/139 |
| Final *R* indices | $R_1$ (I>2σ(I)) = 0.0280; $wR_2$ (I > 2 σ(I)) = 0.0534<br>$R_1$ (all) = 0.0393; $wR_2$ (all) = 0.0564 |
| Largest diff. peak and hole | +3.69 $e^-$/Å$^3$ and -4.48 $e^-$/Å$^3$ |
| R. M. S. deviation from mean | 0.606 $e^-$/Å$^3$ |
| Goodness of fit | 1.044 |

**Table S1b.** Atomic coordinates and isotropic displacement parameters of $Eu_4Bi_6Se_{13}$ single crystal. ($U_{eq}$ is defined as one-third of the trace of the orthogonalized $U_{ij}$ tensor.) Values in parentheses are estimated standard deviations from refinement.

| Atom | Wyck. | Occ. | *x* | *y* | *z* | $U_{eq}$ |
|---|---|---|---|---|---|---|
| Eu1 | 2*e* | 1 | 0.00120(2) | 1/4 | 0.75210(2) | 0.00486(4) |
| Eu2 | 2*e* | 1 | 0.27071(2) | 1/4 | 0.82146(2) | 0.00537(4) |
| Eu3 | 2*e* | 1 | 0.48394(2) | 1/4 | 0.65651(2) | 0.00598(4) |
| Eu4 | 2*e* | 1 | 0.74374(2) | 1/4 | 0.73077(2) | 0.00542(4) |
| Bi1 | 2*e* | 1 | 0.08313(2) | 1/4 | 0.42140(2) | 0.00547(3) |
| Bi2 | 2*e* | 1 | 0.82477(2) | 1/4 | 0.38014(2) | 0.00531(3) |
| Bi3 | 2*e* | 1 | 0.32919(2) | 1/4 | 0.47713(2) | 0.00438(3) |
| Bi4 | 2*e* | 1 | 0.50754(2) | 1/4 | 0.12273(2) | 0.00523(3) |
| Bi5 | 2*e* | 1 | 0.68542(2) | 1/4 | 0.95803(2) | 0.00587(3) |
| Bi6 | 2*e* | 1 | 0.10763(2) | 1/4 | 0.02382(2) | 0.00894(4) |
| Se1 | 2*e* | 1 | 0.37666(3) | 1/4 | 0.33475(3) | 0.00467(8) |
| Se2 | 2*e* | 1 | 0.80043(3) | 1/4 | 0.55944(3) | 0.00549(8) |
| Se3 | 2*e* | 1 | 0.04141(3) | 1/4 | 0.59118(3) | 0.00501(8) |
| Se4 | 2*e* | 1 | 0.56897(3) | 1/4 | 0.48450(3) | 0.00518(8) |
| Se5 | 2*e* | 1 | 0.97929(4) | 1/4 | 0.91020(3) | 0.00949(9) |
| Se6 | 2*e* | 1 | 0.23691(3) | 1/4 | 0.12903(3) | 0.00628(8) |
| Se7 | 2*e* | 1 | 0.85999(3) | 1/4 | 0.23489(3) | 0.00657(8) |
| Se8 | 2*e* | 1 | 0.60715(3) | 1/4 | 0.23850(3) | 0.00490(8) |
| Se9 | 2*e* | 1 | 0.39453(3) | 1/4 | 0.95888(3) | 0.00589(8) |
| Se10 | 2*e* | 1 | 0.11435(3) | 1/4 | 0.27469(3) | 0.00606(8) |
| Se11 | 2*e* | 1 | 0.57888(3) | 1/4 | 0.81538(3) | 0.00510(8) |
| Se12 | 2*e* | 1 | 0.28187(3) | 1/4 | 0.64428(3) | 0.00524(8) |
| Se13 | 2*e* | 1 | 0.80370(3) | 1/4 | 0.06214(3) | 0.00663(8) |

The structure was solved and refined using the Bruker SHELXTL Software Package, using the space group $P2_1/m$, with Z = 2 for the formula unit $Eu_4Bi_6Se_{13}$. The final anisotropic full-matrix least-squares refinement on $F^2$ with 139 variables converged at $R_1$ = 3.93 % for the observed data and $wR_2$ = 5.64 % for all data. The goodness-of-fit was 1.044. The largest peak in the final difference electron density synthesis was 3.69 $e^-/Å^3$, and the largest hole was -4.48 $e^-/Å^3$ with an RMS deviation of 0.606 $e^-/Å^3$. Based on the final model, the calculated density was 7.373 $g/cm^3$ and F (000), 2384 $e^-$.

**Table S2.** Bonding length of Eu under pressure.

| Pressure (GPa) | | 0.37 | 1.21 | 2.5 | 3.3 | 4.4 | 5.7 | 6.7 | 8.9 |
|---|---|---|---|---|---|---|---|---|---|
| Eu1 | Se11 (Å) | 2.621 | 2.063 | 2.006 | 2.450 | 2.018 | 2.050 | 2.059 | 2.197 |
| | Se13 (Å) | >3.8 | >3.8 | >3.8 | >3.8 | >3.8 | >3.8 | >3.8 | 3.297 |
| | Se3 (Å) | 3.270 | 3.349 | 3.277 | 3.163 | 3.317 | 3.262 | 3.199 | 3.178 |
| | Se5 (Å) | 3.102 | 2.873 | 2.900 | 2.740 | 2.838 | 2.794 | 2.79524 | 2.846 |
| | Se1 (Å) | 3.640 | 2.986 | 2.915 | 3.077 | 2.878 | 2.931 | 3.0574 | 2.737 |
| Eu2 | Se3 (Å) | 3.163 | 3.109 | 3.080 | 3.137 | 3.055 | 3.068 | 3.077 | 2.839 |
| | Se9 (Å) | 3.034 | >3.8 | 3.653 | 3.650 | 3.578 | 3.507 | 3.350 | 3.067 |
| | Se8 (Å) | 2.863 | 2.709 | 2.698 | 2.830 | 2.576 | 2.592 | 2.600 | 2.762 |
| | Se13 (Å) | 3.453 | 3.065 | 3.108 | 3.248 | 3.038 | 2.982 | 2.946 | >3.8 |
| | Se7 (Å) | 3.790 | 3.712 | >3.8 | >3.8 | >3.8 | 3.788 | 3.691 | 3.097 |
| Eu3 | Se4 (Å) | 3.334 | 3.007 | 2.912 | 3.038 | 3.062 | 3.034 | 3.039 | 2.753 |
| | Se6 (Å) | 3.120 | 2.886 | 2.977 | 2.958 | 2.812 | 2.770 | 2.730 | 3.062 |
| | Se10 (Å) | 3.583 | >3.8 | 3.733 | 2.723 | 3.479 | 3.426 | 3.329 | 3.349 |
| | Se8 (Å) | 3.515 | 3.665 | 3.465 | 3.653 | 3.569 | 3.576 | 3.562 | 3.493 |
| | Se9 (Å) | 2.661 | 3.032 | 2.919 | 3.548 | 3.066 | 2.961 | 2.947 | 2.784 |
| Eu4 | Se2 (Å) | 3.760 | 2.662 | 2.527 | 2.570 | 2.506 | 2.549 | 2.531 | 2.882 |
| | Se5 (Å) | 3.040 | 3.126 | 3.053 | 3.206 | 3.003 | 3.068 | 3.094 | 3.341 |
| | Se12 (Å) | 3.564 | 3.421 | 3.492 | 3.125 | 3.300 | 3.238 | 3.179 | 3.444 |
| | Se10 (Å) | 3.220 | 3.401 | 3.526 | 3.108 | 3.562 | 3.532 | 3.583 | 3.408 |
| | Se6 (Å) | 3.118 | 3.522 | 3.387 | 3.300 | 3.439 | 3.395 | 3.371 | 3.116 |

The cif files using for PXRD refinement are from previous pressure data.

**Figure S1.** β and volume as a function of temperature and pressure.

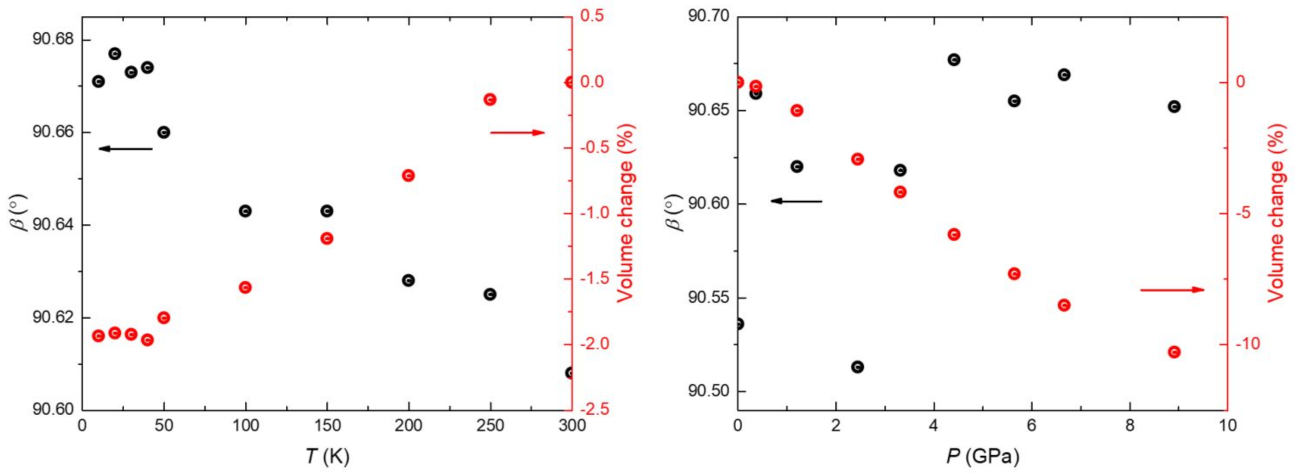

**Figure S2.** $Eu^{3+}$ percentage as a function of volume change.

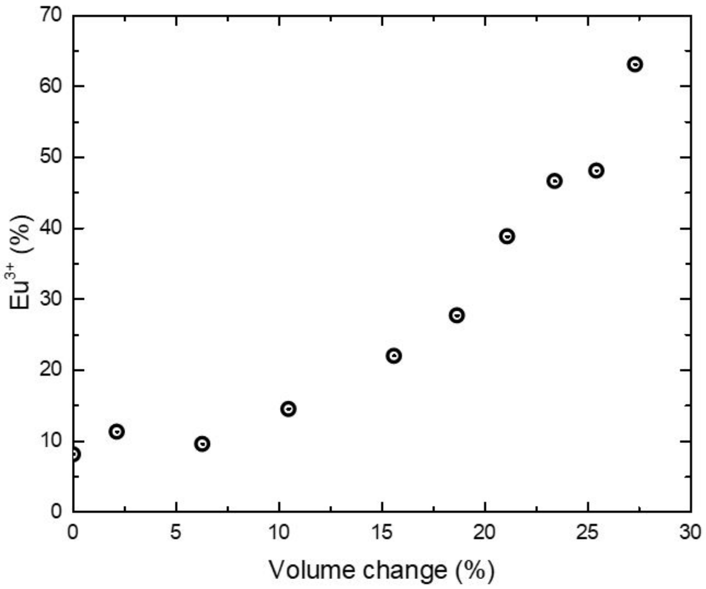


.

**Figure S3.** SEM and EDS results of single crystal $Eu_4Bi_6Se_{13}$. The picture shows the SEM result of the sample, and the table summarizes the EDS results of several spots from two different samples.

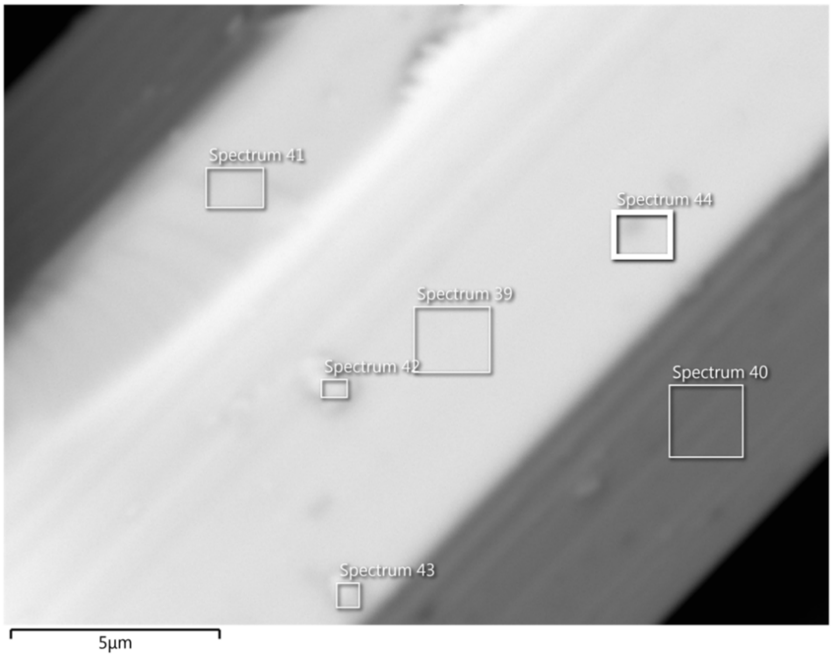


| Elements | At% |
|---|---|
| Eu | 16(1) |
| Bi | 28(1) |
| Se | 56(2) |

**Figure S4.** Magnetization measurements

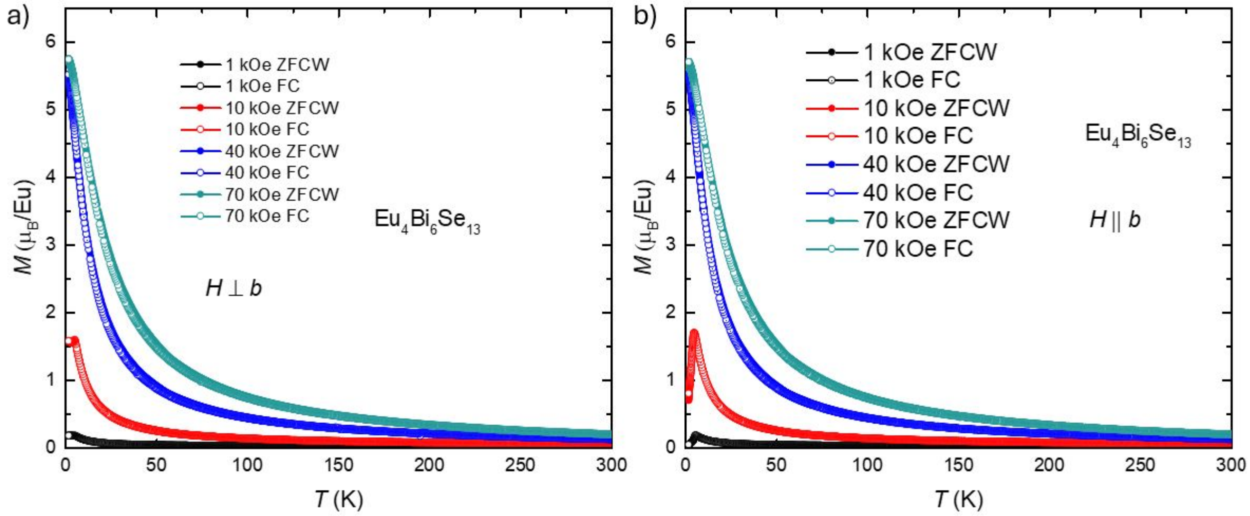


Magnetization measurements as a function of temperature under 1 kOe, 10 kOe, 40 kOe and 70 kOe magnetic field parallel and perpendicular to b-axis.
